# Introduction to collection: confronting the challenges of health research in humanitarian crises

**DOI:** 10.1186/s13031-021-00371-8

**Published:** 2021-05-14

**Authors:** Amit S. Mistry, Brandon A. Kohrt, Blythe Beecroft, Nalini Anand, Iman Nuwayhid

**Affiliations:** 1 Fogarty International Center, U.S. National Institutes of Health, 16A Center Drive, MSC 6710, Bethesda, MD 20892 USA; 2 Division of Global Mental Health, Department of Psychiatry and Behavioral Sciences, George Washington School of Medicine and Health Sciences, Washington, DC USA; 3 Faculty of Health Sciences, American University of Beirut, Beirut, Lebanon

**Keywords:** Humanitarian crisis, Natural disaster, Disease outbreak, Conflict, Displaced populations, Refugees, Research

## Abstract

**Background:**

Humanitarian crises, such as armed conflict, forced displacement, natural disasters, and major disease outbreaks, take a staggering toll on human health, especially in low-resource settings. Yet there is a dearth of robust evidence to inform the governments, non-governmental organizations (NGOs), and other humanitarian organizations on how to best respond to them. The Fogarty International Center of the U.S. National Institutes of Health commissioned a collection of *Research in Practice* articles that highlights the experiences of scientists conducting research in the context of humanitarian crises. Unlike traditional research papers, the case analyses in this collection go beyond *what* research was completed and focus on *why* the research was important and *how* it was conducted in these extremely challenging settings.

**Discussion:**

The papers selected for this collection span 27 countries, cover a broad range of humanitarian crises, and discuss a wide variety of disease and health risk factors. Of the 23 papers in the collection, 17 include an author from the affected country and five papers were authored by humanitarian NGOs. Throughout the collection, 43% of the authors were from low- and middle-income countries.

Across the collection, some general themes emerged that are broadly applicable. Importantly, there is a clear need for more, high-quality research to address evidence gaps. Community engagement, already a key element to global health research, was highlighted as especially important for research involving populations dealing with severe trauma and disruption. Partnership with humanitarian actors, including local governments, local and international NGOs, and UN agencies, was found to be a critical strategy as well.

**Conclusion:**

A variety of audiences will find this collection useful. Global health educators can utilize papers to facilitate discussion around public health practice and equitable partnerships, among other topics. Humanitarian response organizations may use the collection to consider how research may inform and improve their work. Global health researchers, funders, and other stakeholders may use the collection to stimulate dialogue around key scientific research questions and better appreciate the importance of conducting research in humanitarian crises in the context of achieving broader global health goals.

## Background

Hundreds of millions of people around the world are affected by humanitarian crises, such as armed conflict, forced displacement, natural disasters, and major disease outbreaks. Globally, one in six children lives in or near a conflict zone [1] and nearly 80 million people are forcibly displaced from their homes [2]. The frequency, intensity, and complexity of these crises has steadily increased over the past several decades with some countries facing multiple crises at once or dealing with protracted crises that continue for decades [3]. In just the past two years, parts of the world faced severe hurricanes, wildfires, and floods, while armed conflicts continued unabated and millions of refugees remained unable to return home. On top of all of this, the world is simultaneously dealing with the global COVID-19 pandemic.

These crises take a staggering toll on human health, especially in low-resource settings. Yet there is a dearth of robust evidence to inform the governments, non-governmental organizations (NGOs), and other humanitarian organizations responding to them [4]. The Fogarty International Center (FIC) of the U.S. National Institutes of Health (NIH) initiated a project on *Advancing Health Research in Humanitarian Crises* facilitated by and co-chaired by the authors of this paper. The project convened a workshop at NIH in April 2018, which informed an analysis paper and call to action for the global health research community [5]. Through these efforts, the project co-chairs concluded that despite the challenges to conducting research in humanitarian settings, there are examples of high-quality research in the field that should be shared more widely to increase awareness and promote learning.

Accordingly, this collection of *Research in Practice* articles highlights the experiences of scientists conducting research in the context of humanitarian crises. Unlike traditional research papers, the case analyses in this collection go beyond *what* research was conducted and focus on *why* the research was important and *how* it was conducted in these extremely challenging settings. The case analyses respond to these questions by describing the story behind the research and delving into issues specific to the humanitarian setting.

The objectives of this collection of case analyses were to:

1. Highlight common challenges and share strategies for overcoming the difficulties of conducting health research in humanitarian crises in different settings and contexts.
2. Demonstrate the feasibility and importance of health research in humanitarian crises, thereby encouraging researchers and research funders to conduct and support more, high-quality health research in these contexts.
3. Identify scientific areas of high public health significance that can best be addressed through health research in humanitarian settings.

To conceptualize and develop the collection, FIC convened a Steering Committee with representatives from academia from low- and middle-income countries (LMICs) and high-income countries (HICs), a humanitarian NGO, and several NIH Institutes and Centers. A call for proposals was issued in November 2018 and 51 applications were received. The Steering Committee reviewed and scored submissions based on the following selection criteria:

1. Scientific and public health relevance of research question
2. Appropriateness of research approach and design
3. Research conducted in the context of a humanitarian crises
4. Articulation of specific challenges and the strategies used to address them
5. Completeness in responding to the call for proposals
6. Expertise of the submitting team
7. Overall fit for inclusion in the collection

From the submissions, 24 proposals were selected for development into case analysis papers based on scoring from the Steering Committee. Final selection decisions also ensured LMIC and HIC authorship and diversity across geographic locations of studies, types of humanitarian crises (including acute and protracted crises), health and disease areas, and types of research. Each case analysis follows a standardized framework developed by the Steering Committee with input from the editorial staff of *Conflict and Health*, *BMC Public Health*, and the publisher, Springer Nature. Steering Committee members also reviewed drafts of each paper prior to submission for standard peer review by the journals.

For the purposes of this collection, the term “humanitarian crisis” was broadly defined to include: man-made disasters, including armed conflict, forced displacement, and refugee crises; natural disasters, such as floods, earthquakes, droughts, etc.; and major disease outbreaks (e.g. the 2014–2016 Ebola Outbreak in West Africa). “Health research in humanitarian crises” is inclusive of health research conducted in the setting of a humanitarian crisis and/or health research on a population directly affected by a humanitarian crisis (e.g. a refugee population fleeing conflict, relocated to a more stable setting).

The studies described in this collection took place prior to the COVID-19 pandemic. Nevertheless, the experiences described are incredibly relevant as the humanitarian crises currently facing the world become more common, complex, and interrelated.

## Overview of papers

As of May 8, 2021, 18 papers have been published in the collection, with an additional 5 under review. One manuscript was not submitted by the author due to competing demands. Individual papers are published across two journals and a joint landing page for the collection is accessible at https://www.biomedcentral.com/collections/lessonsfromthefield. The landing page will be updated as manuscripts complete the review process.

**Fig. 1:**
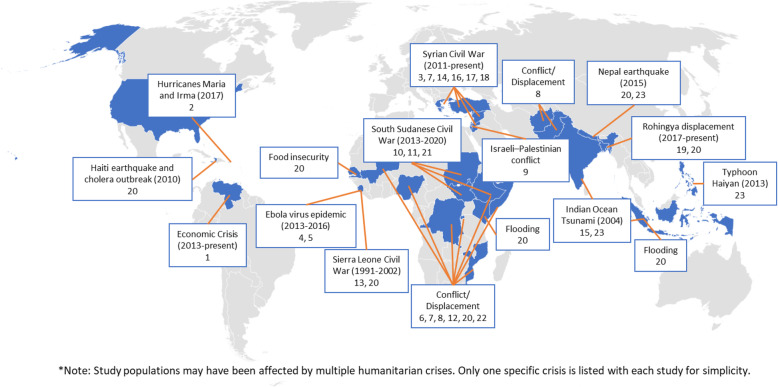
Map of the locations of studies from the collection

Table 1 and Fig. 1 serve as guides to help readers navigate the full collection. As noted in Table 1, the selected case analyses cover humanitarian crises that impact a very broad range of disease and health risk factors. The collection spans acute and protracted crises in 27 countries, as indicated in Fig. 1. Several papers explored a specific area of health in multiple countries on different continents.

**Table 1:** Collection papers by health area

Health Area	Lead Author - Title	Number on Map (Fig. 1)
**Aging**	Maestre, et al. - Research on aging during the Venezuelan humanitarian crisis: the experience of the Maracaibo aging study	1
**Environmental Health**	Vega Ocasio, et al. - Conducting an immersive community-based assessment of post-hurricane experience among Puerto Ricans: lived experience of medical ecology in an environmental disaster and migration	2
**Gender-Based Violence**	Falb, et al. - Pre-positioning an evaluation of cash assistance programming in an acute emergency: strategies and lessons learned from a study in Raqqa Governorate, Syria	3
**Infectious Disease**	Alva, et al. - Conducting mixed-methods research with Ebola survivors in a complex setting in Sierra Leone	4
	*Ho,* et al. *- A Mixed-methods Investigation to Understand and Improve the Scaled-up Infection Prevention and Control in Primary Care Health Facilities During the Ebola Virus Disease Epidemic in Sierra Leone (submitted)*	5
	*Oloruntoba,* et al. *- A case study approach to assess routine childhood immunization coverage of populations in an internally displaced persons (IDP) camp in northeastern Nigeria (submitted)*	6
**Palliative Care**	de Laat, et al. - A case analysis of partnered research on palliative care for refugees in Jordan and Rwanda	7
**Maternal and Child Health**	Gaffey, et al. - Researching the delivery of health and nutrition interventions for women and children in the context of armed conflict: lessons on research challenges and strategies from BRANCH Consortium case studies of Somalia, Mali, Pakistan and Afghanistan	8
	Iellamo, et al. - Breastfeeding knowledge of mothers in protracted crises: the Gaza Strip example	9
	Lasater, et al. - Lessons learned evaluating the baby friendly spaces program for south Sudanese refugees in Gambella, Ethiopia: strengthening research and programmatic partnerships to address maternal and child health and psychosocial needs in humanitarian emergencies	10
	Sami, et al. - An analytic perspective of a mixed methods study during humanitarian crises in South Sudan: translating facility- and community-based newborn guidelines into practice	11
	*Tounkara,* et al. *- A health system intervention in a humanitarian crisis situation in Mali (submitted)*	12
**Mental Health**	Betancourt, et al. - The intergenerational impact of war on mental health and psychosocial wellbeing: lessons from the longitudinal study of war-affected youth in Sierra Leone	13
	*Caman,* et al. *- Mental health research among refugees in Turkey (submitted)*	14
	Padmavati, et al. - Learnings from conducting mental health research during 2004 tsunami in Tamil Nadu, India	15
	Panter-Brick, et al. - Measuring the psychosocial, biological, and cognitive signatures of profound stress in humanitarian settings: impacts, challenges, and strategies in the field	16
	Poole, et al. - A combination sampling approach for epidemiologic research in humanitarian settings: a case analysis of a study of depressive disorder prevalence among refugees in Greece	17
	Weine, et al. *- *Conducting research on building psychosocial support for Syrian refugee families in a humanitarian emergency	18
**Sexual and Reproductive Health**	Ahmed, et al. - Challenges and strategies in conducting sexual and reproductive health research among Rohingya refugees in Cox’s Bazar, Bangladesh	19
**Water, Sanitation and Hygiene (WASH)**	Lantagne, et al. - Lessons learned from conducting six multi-country mixed-methods effectiveness research studies on water, sanitation, and hygiene (WASH) interventions in humanitarian response	20
	*Salih,* et al. *- Efficiency of well-tank-faucet system chlorination to control water-related ailments in a low-resource humanitarian setting in Sudan (submitted)*	21
	Yimer, et al. - Community engagement and building trust to resolve ethical challenges during humanitarian crises: experience from the CAGED study	22
**Multiple Conditions**	Guha-Sapir, et al. - Challenges in public health and epidemiology research in humanitarian settings: experiences from the field	23

More than half of the collection involves forced displacement, conflict, or a combination of the two. Notably, several study populations were affected by more than one crisis simultaneously, such as facing an Ebola outbreak in war-torn Sierra Leone.

An understanding and appreciation for the local context and culture is important in global health and research conducted in humanitarian settings is no exception. All the research featured in this collection was conducted in partnership with local communities, governments, and research institutions based in the countries affected by the crisis. Of the 23 papers, 17 include at least one author from the affected country and seven were led by an author from the affected country. Five of the papers were led by an LMIC-based author and throughout the collection 43% of the authors were from LMICs.

Humanitarian research is often conducted in partnership with humanitarian organizations that are responding to the crisis at hand. In this collection, five papers were authored by humanitarian NGOs and others involved close partnership between local and international NGOs, government actors, and/or UN Agencies.

## Discussion

The case analyses in this collection largely responded to the first two objectives outlined in the Background section of this paper, highlighting the challenges and strategies of conducting research in humanitarian settings (objective 1) and demonstrating the feasibility and importance of this area of research (objective 2). The third objective, identifying scientific areas of high public health significance, was more difficult to realize by the authors and will be the focus of future work.

Readers should note that the collection is not necessarily representative of all research that is conducted in humanitarian settings. Rather, the collection was assembled through a selection process based on specific criteria to present a broad and diverse range of research, enabling readers to draw upon lessons and strategies that are most relevant to their work. Furthermore, the collection does not prescribe guidelines for conducting research in humanitarian settings. However, some general themes have emerged that are broadly applicable.

### Need for research

First and foremost, there is a clear need for humanitarian health research. Several authors noted the limited evidence base in their area of study and all the studies in this collection aimed to address an evidence gap or need. There are many reasons for the limited evidence base in the humanitarian sector. For example, methodological limitations, ethical issues, security concerns, and logistical difficulties were identified as key barriers in the collection. Research teams developed strategies and formed partnerships to address these challenges and ultimately produced meaningful findings. While humanitarian health research is uniquely challenging, it is increasingly important and the evidence gaps in the field cannot be ignored. This collection makes a small contribution to this need, but much more should be done to improve the quantity and quality of research in humanitarian settings.

### Importance of local communities

An important part of nearly every study in this collection was the engagement of affected populations and local communities in the research process. This is an increasingly significant aspect of all health research and becomes especially important when working with people dealing with severe trauma and disruption from a humanitarian crisis. Throughout the collection, different community engagement approaches were used to build trust, navigate ethical issues, and ensure that research findings would benefit communities affected by the crisis.

### Partnership with humanitarian actors

Partnership with humanitarian actors, including local governments, local and international NGOs, and UN agencies, was found to be a critical strategy in the collection. NGOs led the research and writing for several cases in the collection. For many other cases, research was integrated into the response to a humanitarian crisis. Partnerships with humanitarian organizations were critical to addressing logistical and security challenges and improved the likelihood that research findings would be taken up and integrated into humanitarian policies and practices.

## Conclusion: Who can benefit from this collection

The papers in this collection follow a new “Research in Practice” format developed by Springer Nature that is relevant to a broader audience than more traditional research articles.

A key audience for this collection is the global health teaching and learning community. Unique skills are required to navigate challenges that researchers face in humanitarian settings. Our hope is that educators, trainers, and mentors will use this collection to draw examples from as they teach, train, and mentor the next generations of global health and humanitarian health researchers. This collection is ideal for trainees in LMICs as well as trainees studying global health in HIC institutions. The case analysis framework can facilitate discussions about scientific research questions, impact on public health practice, and methods best suited to humanitarian settings. In particular, we hope that this collection can provoke lively discussion among trainees about equitable partnerships in humanitarian research and broader questions of who benefits from humanitarian health research. We hope that the collection can spur dialogue between trainees in high-resource settings and those in regions affected by humanitarian crises. The recent COVID-19 pandemic has demonstrated that the researchers in U.S. and other high-income countries can learn a great deal about humanitarian health research and practice from colleagues in the Global South [6, 7].

In addition, this collection is intended to benefit organizations responding to humanitarian crises, such as governments, NGOs, and international agencies. Non-academic organizations are increasingly leading innovation in assessment of humanitarian health needs and evaluation of interventions. We hope that these real-time innovations will advance research practice and theory for humanitarian health. Our hope is that these organizations can use the collection to consider how research can help inform and improve their work and how their work can advance the feasibility, acceptability, and public health benefit of research. We hope that the collection contributes to strengthening existing partnerships and building new collaborations among humanitarian actors and academic researchers.

Another audience for this collection is the broader global health research community. Our hope is that researchers, funders, and other stakeholders may use the collection to stimulate dialogue around key scientific research questions and better appreciate the importance of conducting research in humanitarian crises in the context of achieving broader global health goals. Efforts focused on humanitarian health research are vital to determine if evidence supports assumptions in the field and to generate novel insights to guide future responses to disasters, war, and pandemics [8]. Humanitarian health research presents unique challenges but should not be treated as an outlier or separate field of study. Rather, it should be considered an integral part of the field of global health.

## Data Availability

Not applicable.

## Abbreviations

FIC: Fogarty International Center
HIC: High-income countries
IDP: Internally displaced persons
LMIC: Low- and middle-income countries
NGO: Non-governmental organizations
NIH: U.S. National Institutes of Health

## References

[1] Bahgat K, Dupuy K, Østby G, Rustad SA, Strand H, Wig T (2018). Children and armed conflict: what existing data can tell us.

[2] United Nations High Commissioner for Refugees (UNHCR) (2020). UNCHR Figures at a Glance.

[3] United Nations High Commissioner for Refugees (UNHCR). Global Trends: Forced Displacement in 2019. Geneva: UNHCR; 2020. https://www.unhcr.org/globaltrends2019/.

[4] Blanchet K, Ramesh A, Frison S, Warren E, Hossain M, Smith J (2017). Evidence on public health interventions in humanitarian crises. Lancet.

[5] Kohrt BA, Mistry AS, Anand N, Beecroft B, Nuwayhid I (2019). Health research in humanitarian crises: an urgent global imperative. BMJ Glob Health.

[6] Kola L, Kohrt BA, Hanlon C, Naslund JA, Sikander S, Balaji M (2021). COVID-19 mental health impact and responses in low-income and middle-income countries: reimagining global mental health. Lancet Psychiatry.

[7] Kohrt BA. What can the USA learn from the mental health response to COVID-19 in low-income and middle-income countries? Lancet Psychiatry. 2021; In press.

[8] Tol WA, Ager A, Bizouerne C, Bryant R, El Chammay R, Colebunders R (2020). Improving mental health and psychosocial wellbeing in humanitarian settings: reflections on research funded through R2HC. Conflict Health.

