# Correction to: Introduction to collection: confronting the challenges of health research in humanitarian crises

**DOI:** 10.1186/s13031-021-00383-4

**Published:** 2021-06-17

**Authors:** Amit S. Mistry, Brandon A. Kohrt, Blythe Beecroft, Nalini Anand, Iman Nuwayhid

**Affiliations:** 1 Fogarty International Center, U.S. National Institutes of Health, 16A Center Drive, MSC 6710, Bethesda, MD 20892 USA; 2 Division of Global Mental Health, Department of Psychiatry and Behavioral Sciences, George Washington School of Medicine and Health Sciences, Washington, DC USA; 3 Faculty of Health Sciences, American University of Beirut, Beirut, Lebanon

**Correction to: Confl Health 15, 38 (2021)**

**https://doi.org/10.1186/s13031-021-00371-8**

The original publication of this article [1] was missing the following disclaimer:

- The findings and conclusions in this article are those of the authors and do not necessarily represent any official position or policy of the U.S. National Institutes of Health, the U.S. Department of Health and Human Services, or any other institutions with which authors are affiliated.

The original article has been updated.
